# Risks and features of secondary infections in severe and critical ill COVID-19 patients

**DOI:** 10.1080/22221751.2020.1812437

**Published:** 2020-09-08

**Authors:** Haocheng Zhang, Yi Zhang, Jing Wu, Yang Li, Xian Zhou, Xin Li, Haili Chen, Mingquan Guo, Shu Chen, Feng Sun, Richeng Mao, Chao Qiu, Zhaoqin Zhu, Jingwen Ai, Wenhong Zhang

**Affiliations:** a Department of Infectious Disease, Huashan Hospital affiliated to Fudan University, Shanghai, People’s Republic of China; b Department of Laboratory Medicine, Shanghai Public Health Clinical Center, Shanghai, People’s Republic of China

**Keywords:** COVID-19, secondary infection, intensive care, SARS-CoV-2, respiratory ventilations, intravascular devices

## Abstract

*Objectives* Severe or critical COVID-19 is associated with intensive care unit admission, increased secondary infection rate, and would lead to significant worsened prognosis. Risks and characteristics relating to secondary infections in severe COVID-19 have not been described. *Methods* Severe and critical COVID-19 patients from Shanghai were included. We collected lower respiratory, urine, catheters, and blood samples according to clinical necessity and culture and mNGS were performed. Clinical and laboratory data were archived. *Results* We found 57.89% (22/38) patients developed secondary infections. The patient receiving invasive mechanical ventilation or in critical state has a higher chance of secondary infections (*P*<0.0001). The most common infections were respiratory, blood-stream and urinary infections, and in respiratory infections, the most detected pathogens were gram-negative bacteria (26, 50.00%), following by gram-positive bacteria (14, 26.92%), virus (6, 11.54%), fungi (4, 7.69%), and others (2, 3.85%). Respiratory Infection rate post high flow, tracheal intubation, and tracheotomy were 12.90% (4/31), 30.43% (7/23), and 92.31% (12/13) respectively. Secondary infections would lead to lower discharge rate and higher mortality rate. *Conclusion* Our study originally illustrated secondary infection proportion in severe and critical COVID-19 patients. Culture accompanied with metagenomics sequencing increased pathogen diagnostic rate. Secondary infections risks increased after receiving invasive respiratory ventilations and intravascular devices, and would lead to a lower discharge rate and a higher mortality rate.

## Introduction

COVID-19 is a newly recognized illness that has spread rapidly throughout Wuhan to the whole world. As of July 12th, total number of cases has risen to over 12 million globally that is a near exponential growth, of them about 6.7% of patients deceased [1]. Around 20% of SARS-CoV-2 infection would progress to severe or critical state and lead to significant worsened prognosis. Previous studies demonstrated severe SARS-CoV-2 pneumonia was associated with intensive care unit admission, increased secondary infection rate, and higher risk of invasive procedures. Up until now, risks and characteristics relating to secondary infections in severe COVID-19 have not been described.

In other SARS and MERS epidemics, patients receiving invasive mechanical ventilation were easily developed secondary infections and had higher mortality. Studies showed that there was a sharp increase in MRSA, Stenotrophomonas, and Candida species infectious rate among SARS ICU patients [2]. Therefore, bacteria secondary infection might be a key element that promoted severe disease and mortality [3]. First study targeted on 52 critically ill patients with SARS-CoV-2 pneumonia demonstrated that hospital-acquired infection occurred in seven (13.5%) patients and finally four of them deceased [4]. A recent COVID-19 study focusing on deceased patients showed sepsis (113/113;100%) acted as one of the main complications [5], indicating that secondary infection is of great importance to prognosis and subsequent treatment of COIVD-19 patients.

In our study, we analysed samples of critical and severe patients by culture and mNGS according to clinical necessity, and aimed to describe risks of secondary infection, multiple infectious sites, occurrence time, and incident rate of infection related to clinical invasive operation.

## Materials and methods

### Patients

We performed a retrospective, multi-centre cohort study and included severe and critical COVID-19 patients from 22nd January to 30th April admitted to Shanghai Public Health Clinical Center and Guanggu District, Tongji Hospital, Tongji Medical College, Huazhong University of Science and Technology. COVID-19 cases were diagnosed according to WHO interim guidelines. All patients received a positive result of RT–PCR tests targeting SARS-CoV-2.

Severe COVID-19 cases are defined [6] as any one of the following: (1) Respiratory distress, respiratory rates ≥30 per minute; (2) Pulse oxygen saturation ≤93% on room air (3) PaO2/FiO2 ≤ 300 mmHg. Definition [6] of critical cases satisfied any one of the followings: (1) Respiratory failure where invasive ventilation is necessary (2) Signs of shock (3) Failure of any other organ where ICU care is necessary.

Secondary infection was diagnosed when the patients had clinical symptoms presenting infections or positive radiologic evidence, and a positive laboratory-confirmed aetiologic result (culture positive or mNGS positive confirming by RT–PCR) after 48 h of admission. The final diagnosis of causative agents was made according to the clinical physician expert groups’ discussion results. Then, we recorded clinical and epidemiologic data and followed-up outcomes. Written consents were obtained from the patients or their family members.

### Sample procedures

Samples collected from patients included lower respiratory samples, urine, catheters, and serum for aetiology examinations. Lower respiratory (sputum, bronchoalveolar lavage fluid, and endotracheal aspirate) and urine samples were sent every 2–3 days since admission. Endotracheal aspirate was collected from patients with invasive mechanical supports (tracheal intubation, tracheotomy). BALF was sent for test when patients accepted bronchoscopy operation. Blood culture was examined for suspected sepsis patients with a high temperature suggested by Shanghai expert consensus statement [7]. All suspected sepsis patients with indwelling catheters were examined simultaneously with peripheral venous and catheter blood culture.

Metagenomic next-generation sequencing (mNGS) was performed when clinically necessary. All the patients met the criteria of critical COVID-19 were considered to get mNGS test of blood and sputum samples when they were suspicious for secondary infection (when they had clinical symptoms presenting infections or positive radiologic evidence) 48 h after admission. To assist diagnosis pathogenic microbes, extracted RNA sample was sent for high-throughput mNGS [8], which was sequenced on Illumina Nextseq platfrom using a single-end 75 bp (SE75) strategy. Low-quality reads and reads derived from human genome sequences were removed. The filtered reads were then aligned to a created database for taxonomic classification, and finally pathogen was identified [9].

### Statistical analysis

We compared categorical variables using Chi-square test. Continuous variables were analysed with Mann–Whitney *U* test among groups. All statistical analyses were performed using Stata 14.0 or GraphPad 8.0. For comparisons, a *P-*value less than 0.05 was considered statistically significant.

## Results

### Baseline characteristics

We included all 612 patients from Shanghai Public Health Clinical Center and Guanggu District, Tongji Hospital, Tongji Medical College, Huazhong University of Science and Technology from 22nd January to April 30th (Supplementary Figure). A total of 38 severe and critical COVID-19 patients were included, including 14 severe cases and 24 critical cases. The average age of enrolled patients was 64.76 years (SD 13.76) with 30 (78.95%) patients over 60 years old. 32 (84.21%) patients were male, and chronic diseases were noted in 24 (63.16%) patients. Neither the baseline leucocytes account nor inflammatory factors were different between secondary infection and non-infection patients. The computerized tomography (CT) or X-ray of most patients showed ground glass density, thickened lobular septa, and “paving stone”-like change (Supplementary Tables 1,2).

### Secondary infections in severe and critical COVID-19 cases

Overall, 22 (57.89%) patients developed secondary infections, among which, 21, 13, and 7 patients had respiratory, blood-stream and urinary infections, respectively(Supplementary Table 3). Secondary-infection rate in critical patients is much higher than that in severe patients (83.33% (20/24) vs 14.29% (2/14), *P*<0.0001).

### Profiles of pathogens in different types of secondary infections

**Figure 1.**
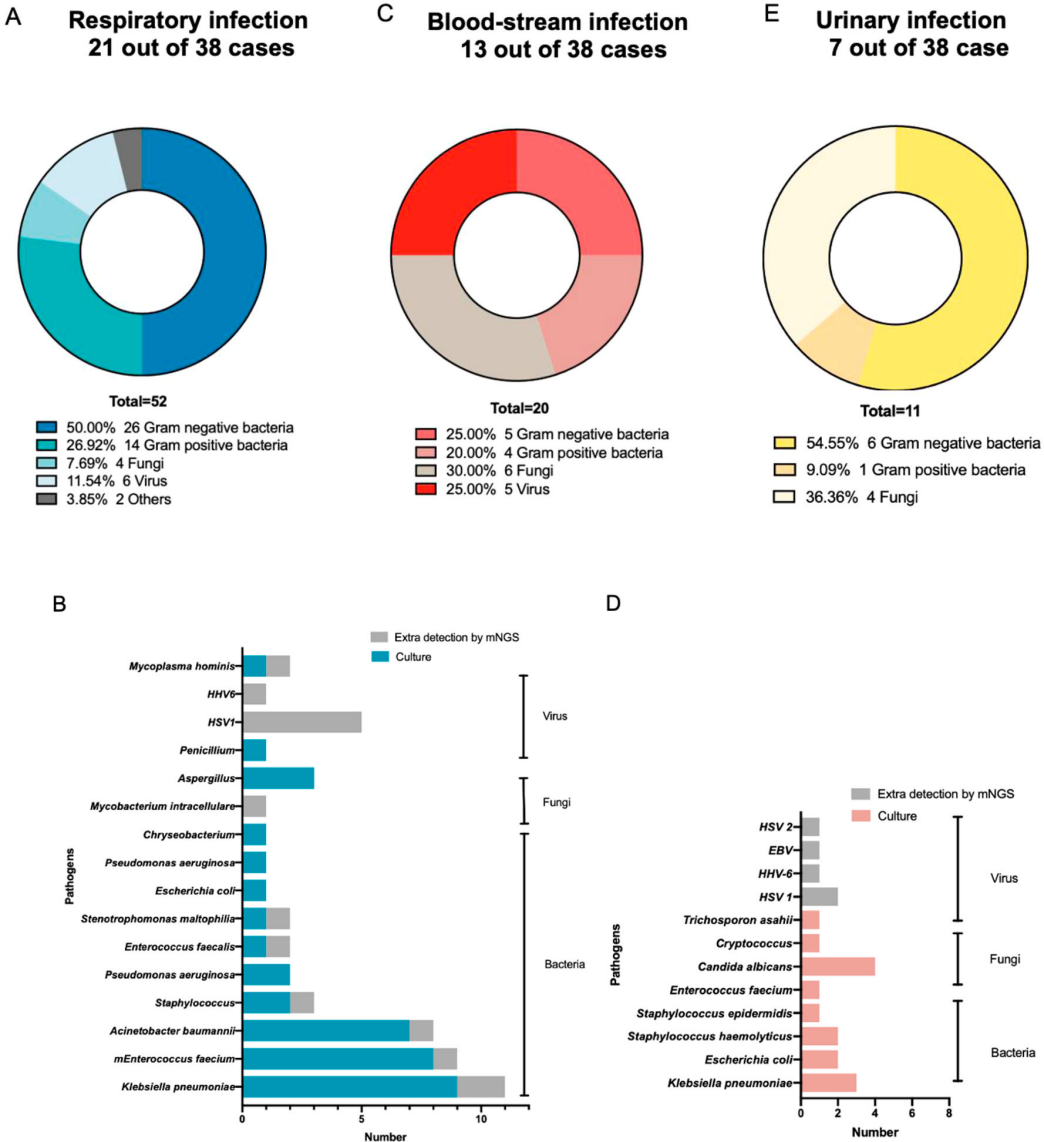
Proportions and distributions of pathogens detected in different secondary infections. (A) Composition of pathogens in respiratory secondary infections. (B) Distributions of pathogens detected in respiratory secondary infections. The blue stripe indicated bacteria and fungi reported by culture, while grey stripe showed pathogens extra detected by mNGS. (C) Composition of pathogens in blood-stream secondary infections. (D) Distributions of pathogens detected in blood-stream secondary infections. The red stripe indicated bacteria and fungi reported by culture, while grey stripe showed pathogens extra detected by mNGS. (E) Composition of pathogens in urinary secondary infections.

A total of 52 pathogens were confirmed (Figure 1(A)) in respiratory infection patients, most of which were gram-negative bacteria (26, 50.00%), following by gram-positive bacteria (14, 26.92%), virus (6, 11.54%), fungi (4, 7.69%) and others (2, 3.85%). Most common pathogens encompassed *Klebsiella pneumoniae* (11), *Enterococcus faecium* (9), *Acinetobacter baumannii* (8), *HSV1*(5), etc. (Figure 1(B)).

Extra detection ability of mNGS mostly exhibited in virus and all of them belonged to *Herpesviriade,* consisting of *HSV1* and *HHV6*. Some common nosocomial infection bacteria including *Klebsiella pneumoniae, Acinetobacter baumannii, Enterococcus spp*., etc*.* were also detected. All of the patients with viremia were critical COVID-19 patients.

We categorized secondary respiratory infections of COVID-19 into 8 types (Table 1). Six of 21 patients had virus, gram-positive bacteria and gram-negative bacteria at the same time. Two patients only infected by fungi and 7 by gram-negative bacteria. No COVID-19 patients had fungi, virus, gram-positive bacteria, and gram-negative bacteria secondary infection simultaneously. All patients with blood-stream infections were classified as critical cases. All 13 blood-stream infectious patients had respiratory infections as the same time. A total of 20 pathogens were detected in 13 patients with blood-stream infections (Figure 1(C)). 30.00% (6/20) proportion of pathogens were fungi, while virus and bacteria accounted for 25.00% and 30.00%, respectively. Most detected pathogens were *Candida albicans* (4). The viruses included *HSV1, HSV2, EBV*, and *HHV6* (Figure 1(D)). In urinary secondary infections, 7 pathogens from 7 patients were illustrated, including *gram-negative bacteria* (6) and *Enterococcus faecium* (1) (Figure 1(E)).

**Table 1.** Secondary infection types of respiratory infections.

Secondary infection types	Number of patients (*n*)
Fungi	2
Gram-positive bacteria	1
Gram-negative bacteria	7
Gram-positive bacteria and gram-negative bacteria	2
Fungi, gram-positive bacteria and gram-negative bacteria	1
Virus, Gram-positive bacteria and gram-negative bacteria	5
Fungi, virus and gram-positive bacteria	1
Combined with other pathogens*	2

*Patient8 with *Mycobacterium intracellulare* and patient 10 with *Mycoplasma hominis* were classified as “Combined with other pathogens”.

### Secondary infections associated with invasive supportive therapy

We further evaluated the risks of secondary infections undergoing invasive supports in severe COVID-19 patients. All enrolled patients received different respiratory supportive therapy, 31 out of which experienced high flow nasal cannula (HFNC). Tracheal intubation and tracheotomy were performed on 23 and 14 patient, and 8 patients using extra corporeal membrane oxygenation (ECMO). We next calculate the occurrence time of respiratory infection after the above-mentioned supportive therapy. A proportion of 12.90% (4/31) patients were infected after HFNC and a total of 6 pathogens were detected. After tracheal intubation and tracheotomy, infection rate raised to 30.43% (7/23) and 92.31% (12/13) with 14 and 31 pathogens detected, respectively. The median time of infection after HFNC, tracheal intubation, and tracheotomy was 7.5, 4.5, and 9 days after clinical operations, respectively (Table 2(a)).

**Table 2.** Profiles and detected pathogens following respiratory supportive strategies and invasive operations.

	High flow	Tracheal intubation	Tracheotomy
Cases (*n*)	31	23	14
Perform time (d, mean ± SD)	7.58 ± 5.12	9.04 ± 5.92	15.64 ± 7.03
Proportion of infection (%)	12.90% (4/31)	30.43% (7/23)	92.31% (12/13)
Occurrence time of infection in patients (median (range))	7.5 (1–22)	4.5 (1–19)	9 (1–26)
Detected respiratory pathogens (*n*)	6	14	31

	IVC	ECMO	CRRT
Cases (*n*)	25	8	10
Perform time (d, mean ± SD)	8.76 ± 6.55	14.88 ± 9.20	19.2 ± 14.28
Proportion of infection (%)*	52.00% (13/25)	75.00% (6/8)	60.00% (6/10)
Occurrence time of infection in patients (median (range))	12 (1–35)	12 (1–21)	10.5 (2–23)
Detected respiratory pathogens (*n*)	19	9	10

*Some patients received more than one intravascular device. If the pathogen appeared after all the received intravascular device, this pathogen would be calculated into two or three groups.

**Figure 2.**
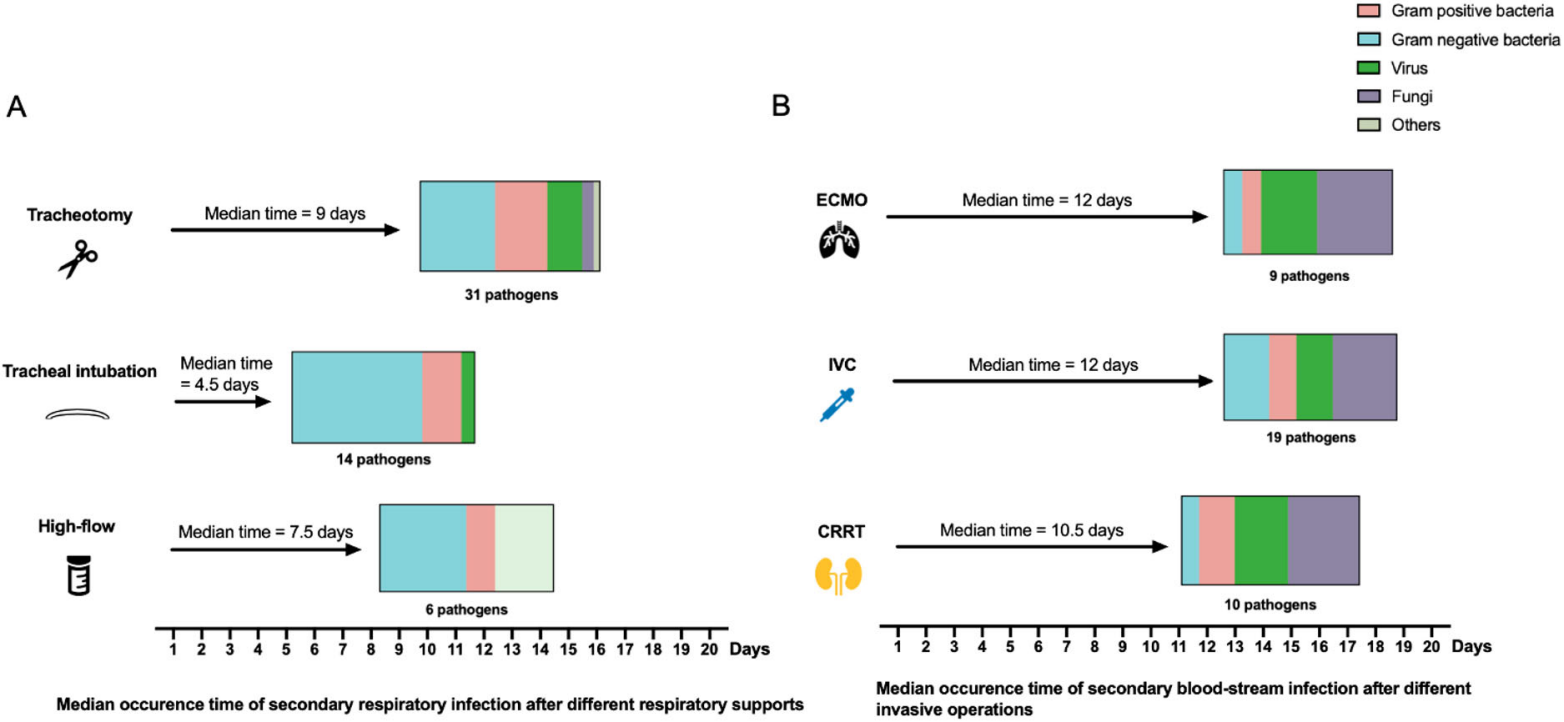
Detection of secondary respiratory and blood-stream infection pathogens following clinical operations. (A) The median time indicated the infection period after accordingly respiratory support. (B) The median time indicated the infection period after different intravascular devices. *P-*value of median time was calculated and annotated on the right.

What’s more, we analysed compositions of respiratory tract pathogens and differences in median occurrence time of respiratory supportive therapy. Severe and critical patients tend to develop secondary infections after 7.5 (1–22) days of HFNC, and bacteria accounts for 66.67% (4/6) in detected pathogens. No viral infections were detected after HFNC (Table 2(a)). After 4.5 (1–19) days of tracheal intubation, patients started to present with secondary infections and gram-negative bacteria accounted for 71.43% (10/14). For tracheotomy, infections tended to take more time to occur than that of tracheal intubations and a total of 31 pathogens were detected, including 13 gram-negative bacteria, 9 gram-positive bacteria. 6 viruses, 2 fungi and *Mycobacterium intracellulare* (Figure 2(a)).

We next performed analyses of compositions of blood-stream infection pathogens and differences in median occurrence time after invasive operations. As shown in Table 2(b), the median time of infection after IVC (intravenous catheter*)*, ECMO, and CRRT (continuous renal replacement therapy) was 12, 12, and 10 days, respectively. A total of 19 pathogens were detected after IVC, of which 5 pathogens were gram-negative bacteria, 3 were gram-positive bacteria, 4 were viruses and 7 were fungi. Fungi accounted for 44.44% (4/9) and 40.00% (4/10) after ECMO and CRRT operations separately.

### Analyses of outcomes and discharges

**Figure 3.**
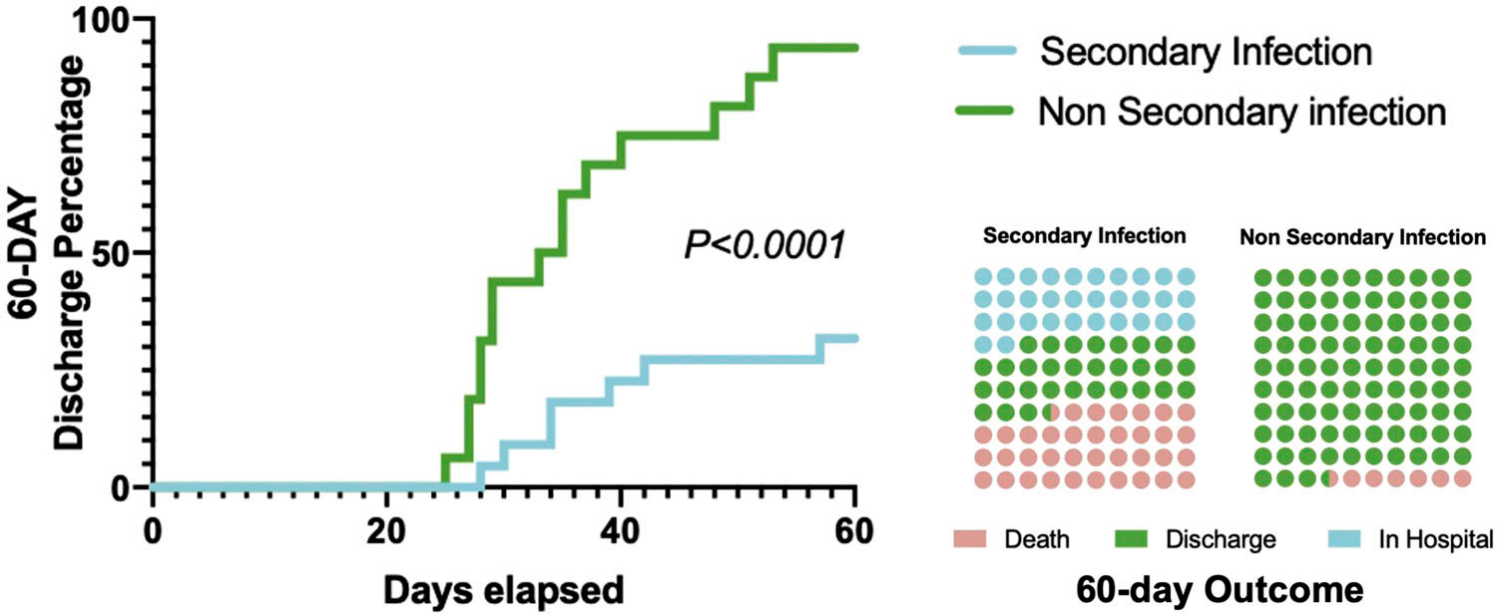
The outcome of COVID-19 patients with or without secondary infection.

Patients without secondary infection had a significantly higher 60-day discharge rate improvement different from patients with secondary infection (*P*<0.001). Among 16 non-infection patients with severe and critical SARS-CoV-2 infection, 15 (93.75%) patients had discharged from the hospital within 60 days, and the median duration from ICU admission to discharge was 31 (IQR 27–39) days. 8 (36.36%) of 22 secondary infection patients had died by 60 days. Compared with non-infection patients, patients with secondary infection were more likely to receive invasive mechanical ventilation (86.36% (19/22) vs 25.00% (4/16), *P* < 0.0001). (Figure 3).

## Discussion

COVID-19 is a newly emerging life-threatening infectious diseases and we are still trying to rapidly increase our knowledge concerning the disease. According to a study that enrolled 72,314 cases in China, the overall mortality rate of the confirmed cases was 2.3%, while in critical patients, the mortality rate would sharply rise to around 50%. Therefore, to further lower the fatality in critical COVID-19 cases is crucial. While Secondary infection is a neglectable question in severe and critical COVID-19 patients. Yang et al. targeted 52 critically ill patients with SARS-CoV-2 pneumonia in Wuhan and demonstrated that hospital-acquired infection occurred in seven (13.5%) patients and finally four of them deceased [4]. Zhou et al. reported a 50% secondary infection rate in non-survivors (27/54) and ventilator-associated pneumonia occurred in 31% (10/32) patients requiring invasive respiratory support [10].

The underlying pathogenesis of secondary infection in severe and critical COVID-19 cases lied in the interactions of host and pathogens [3,11], including virulence of pathogens, dysregulations of immune responses, and disturbed microbiota during viral pneumonia. Virus pneumonia and secondary infection acted as mutually reinforcing factors to promote the progression of COVID-19. Severe SARS-CoV-2 infection caused multiple damages in the lungs, which can largely decrease the oxygen and carbon dioxide diffusion capacities. The disruption of surfactant and the sloughing of cells into the airways may provide access and a rich source of nutrients, promoting rapid bacterial growth [12]. Both impact of microbiome change and bacteria virulence factor can alter the immune responses to SARS-CoV-2, resulting in the rebound of viral titre [13] and high mortality in severe and critical patients.

In this study, we reported three types of secondary infections in severe and critical COVID-19 patients. A total of 21, 13, and 7 patients were finally diagnosed with respiratory, blood-stream, and urinary infections.

In respiratory infections, bacterial pneumonia ranked first, including *E.faecium*, *A.baumannii*, *K.pneumoniae*, etc. This pathogen distribution was partially similar to other COVID-19 studies in which several researchers have reported common pathogens including *Acinetobacter baumannii*, *Klebsiella pneumoniae*, *Candida*, etc. in severe COVID-19 cases [14]. The reason behind this pathogen distribution may be that most of severe and critical cases were in ICU and underwent invasive procedures, which may increase the chance of hospital-acquired infection. For example, a higher proportion of gram-positive bacteria was observed after tracheotomy and it might correlate with skin colonization during operations.

One study in the USA recently has revealed that mortality of COVID-19 patients receiving invasive mechanical ventilation (IMV) were as high as 88.1% [15], significantly higher than those who did not receive mechanical ventilation. This result was similar to those previously published in China [10], and the reasons lying behind included the overall critical state of the patients who are on IMV and the increased risk of complications accompanied with lengthened ICU stay and IMV usage. Although IMV might increase the risk of secondary infections, many guidelines, including NIH and the National Health Commission of China guideline, has recommended the invasive mechanical measures as the first priority in the treatment of critical COVID-19. Previous study compared a conservative oxygen strategy (target SpO2 88% to 92%) to a liberal oxygen strategy (target SpO2 ≥ 96%) [16]. Mortality increased by 8% and 14% among those who received the lower targeted oxygen level at Day 28 and Day 90, respectively. Therefore, respiratory support was one of the key treatment strategies in severe COVID-19 infections [17] for decreasing the mortality, but timely detection and treatment of secondary infections should also be emphasized for the improved prognosis.

We noted 34.21% (13/38) of patents developed blood-stream infections (BSI) and found IVC, respiratory infection, and urinary infection accounted for the main causes of BSI. Our results were supported by the previous study demonstrating that intravascular devices (52%), and urinary tract infection (18%), and pneumonia (16%) were the most common infectious source of BSI in nosocomial infection [18]. Interestingly, we found *Candida albicans* was the top detected pathogen in blood-stream infection. Such opportunistic colonization pathogen has become commonly detected causative agents in severe and critical COVID-19 status, which may be explained by that those patients were in immunocompromised status because of viral sepsis. Previous study showed that sustained and substantial reduction of the peripheral lymphocyte counts, especially CD4 T and CD8 T cells, is the representative of immune suppression stage after the cytokine storm activation phase [19]. The dysregulated immune response may be associated with a high risk of developing secondary bacterial infection.

We reported a bit higher secondary infection rate of 57.89% (22/38) than that in previous data ranging from 13.5% to 31% [4,10]. Apart from pulmonary infection reported in previous studies, we also analysed blood-stream infection and urinary infections. In addition, we sent respiratory, blood, and urinary samples timely and performed mNGS for extra detection. We suppose the higher secondary infection rate might lie in patient characteristics (many of our patients are critically ill or in severe state), repeated culture of multiple samples, and extra detection of viruses and other pathogens through mNGS. Notably, we found mNGS showed a higher detection rate of secondary infections, which had an advantage mainly in unculturable viral infection and some nosocomial infection neglected by clinical culture. According to the mNGS results of respiratory and blood samples, 25.00% of the patients changed the drug selection.

Our studies have several limitations. First, we performed a single-centre retrospective study with a relatively small sample size. More studies are needed to further validate our findings in the future. Second, mNGS was carried out according to clinical necessity in our study thus some patients did not receive mNGS tests. Third, antimicrobial susceptive tests were only taken for part of pathogens including *Acinetobacter baumannii*, *Klebsiella pneumoniae*, and *Pseudomonas aeruginosa*. A full-spectrum antibiotic profile should be analysed later.

## Conclusion

Our study originally illustrated the proportion of secondary infections in severe and critical ill COVID-19 patients. Traditional culture accompanied with metagenomics sequencing will increase the pathogen diagnostic rate. Patients tend to have secondary infections after receiving invasive respiratory ventilations. More than half of the detected respiratory pathogens were bacteria while virus and fungi accounted for a high proportion of in blood-stream infections. After two-month follow-up, we found secondary-infection patients had a lower discharge rate and a higher mortality rate than without secondary infections.

## Declaration of interest

All authors report no potential conflict of interest.

## Supplementary Material

0712-EMI-Supplementary_Table.docx

Revison_letter.docx

## Abbreviations

**Abbreviations:** BALF: bronchoalveolar lavage fluid; BSI: Blood-stream infection; CRRT: continuous renal replacement therapy; COVID-19: coronavirus disease 2019; CT: Computerized Tomography; ECMO: extracorporeal membrane oxygenation; HFNC: high flow nasal cannula; ICU: intensive care unit; mNGS: metagenomic next-generation sequencing; IVC: intravenous catheter; MRSA: Methicillin-resistant *Staphylococcus aureus*; RT–PCR: real-time reverse transcriptase polymerase chain-reaction; SARS-CoV-2: severe acute respiratory syndrome coronavirus 2
