# Supplementary material for: Risks and features of secondary infections in severe and critical ill COVID-19 patients
Source: Emerg Microbes Infect. 2020 Sep 8;9(1):1958–64. doi: 10.1080/22221751.2020.1812437 (PMC8284966; doi:10.1080/22221751.2020.1812437)
Supplement: 0712-EMI-Supplementary_Table.docx [file TEMI_A_1812437_SM3314.docx]

**Supplementary Table1. Basic characteristics**

|  | **Secondary infection（22）** | **Non-secondary infection（16）** | ***P*** |
| --- | --- | --- | --- |
| **Invasive Mechanical Ventilation** | 19 | 4 | 0.000 |
| **Critical status** | 20 | 4 | 0.000 |
| **Sex (male)** | 17 | 15 | 0.370 |
| **Comorbidities*** | 14 | 10 | 0.604 |
| **Age, years** | 68.36$\pm12.07$ | 59.81$\pm14.75$ | 0.027 |
| **White blood cells, × 10^9^/L** | 6.95$\pm4.00$ | 5.09$\pm$2.28 | 0.079 |
| **Lymphocytes, × 10^9^/L** | 4.29$\pm3.48$ | 1.84$\pm$1.21 | 0.005 |
| **C-reaction protein, g/L** | 85.32$\pm61.13$ | 90.29$\pm68.93$ | 0.820 |
| **Erythrocyte sedimentation rate, mm/L** | 57.71$\pm$33.66 | 65.68$\pm30.24$ | 0.460 |
| **Procalcitonin, mg/L** | 0.37$\pm$0.63 | 0.14$\pm$0.20 | 0.108 |

*Comorbidities: Hypertension, diabetes, cerebrovascular diseases, chronic obstructive pulmonary diseases,

cardiovascular disease, tumor, chronic hepatitis diseases.

**Supplementary Table2. The pulmonary CT or x-ray features of patients**

|  | CT or X-ray findings |
| --- | --- |
| Patient 1 | Right lower lobe, upper left lobe, left lower lobe showed scattered patch-like ground glass density shadows, some lesions were accompanied by thickened lobular septa, some showed "paving stone"-like changes and bronchial inflation Sign |
| Patient 2 | flakes and large ground glass-like density increase were seen in the field of the two lungs, |
| Patient 3 | The upper lobe of the right lung, the middle lobe, the lower lobe of the right lung, and the upper and lower lobes， some lesions were accompanied by thickened lobular septa, some showed "paving stone"-like changes and bronchial inflation Sign |
| Patient 4 | Right upper lobe， Right lower lobe， Left lower lobe，and Left upper lobesome lesions were accompanied by thickened lobular septa, some showed "paving stone"-like changes and bronchial inflation Sign. |
| Patient 5 | The upper, middle and lower lobes of the right lung and the upper and lower lobes of the left lung are scattered with patchy ground glass density shadows, mainly distributed in the dorsal subpleural area, with the thickened leaflet interval in the area, and some of them are changed as "paving stones" |
| Patient 6 | There were multiple patchy ground glass density shadows with partial consolidation under the upper pleura of the two lungs, and a few patchy ground glass density shadows in the right lung midfield. In the lower lobe of the lungs, there were multiple lamellar consolidations and fibrous cord shadows, in which air bronchial signs were seen; the left lower lobe bronchus was slightly dilated, and there was no obvious abnormality in the bronchial wall. |
| Patient 7 | In the upper and lower lobes of the right lung, and the upper and lower lobes of the left lung, scattered patch-like ground glass density shadows were seen. Some lesions are accompanied by thickened lobular interval, some are changed in the shape of ‘paving stone’, the boundary is not clear, and some see signs of inflatable bronchi |
| Patient 8 | The right upper lobe, the right middle lobe, the right lower lobe, and the left upper lobe and the left lower lobe showed scattered patch-like ground glass density shadows. Some lesions were thickened with lobular septa,with inflatable Bronchial Sign |
| Patient 9 | Coronary virus pneumonia was diffuse in both lungs, and there were diffuse patchy, ground glass-like exudative solid changes in both lungs, and the border was unclear. |
| Patient 10 | The upper, middle and lower lobes of the right lung and the upper and lower lobes of the left lung were scattered with patch-like ground glass density shadows. Some lesions are accompanied by thickened lobular septa, some are ‘paving stone’-like, and some have airy bronchial signs. Pleural effusions on both sides |
| Patient 11 | A small amount of pleural effusion in the pleural cavity; the upper, middle and lower lobes of the right lung, and the scattered glazed glass density shadows in the upper and lower lobes of the left lung. Some lesions are accompanied by thickened lobular septa, some are ‘paving stone’-like, and some have signs of inflatable bronchi |
| Patient 12 | Diffuse coronavirus pneumonia in both lungs with focal fibrosis, and a small amount of fluid in the left pleural cavity. The right upper lobe, middle lobe, right lower lobe, left and upper lobe of the left lung are scattered in patchy ground glass shadows, some lesions are thickened with lobular septa, some are "paving stone" changes, and some have inflatable bronchi |
| Patient 13 | Right upper lobe, right middle lobe, right lower lobe, left lower lobe, left lower lobe, left lower lobe, scattered patchy ground glass density shadow, some lesions with thickened lobular septa, some showed "paving stone"-like changes, and some were inflated Bronchial sign |
| Patient 14 | Right upper lobe, right middle lobe, right lower lobe, left lower lobe, left lower lobe, left lower lobe, scattered patchy ground glass density shadow, some lesions with thickened lobular septa, some showed "paving stone"-like changes, and some were inflated Bronchial sign |
| Patient 15 | Right upper lobe of right lung Middle lobe of right lung Lower lobe of right lung Left lobe of left lung See scattered patch of ground glass density shadow, some lesions with thickened lobular septa, some appear "paving stone"-like changes, some see inflation bronchial signs |
| Patient 16 | Coronary viral pneumonia in both lungs; patchy ground-glass density scattered scattered under the pleura of both lungs. Some lesions are accompanied by thickened lobular septa, and some are changed in the shape of ‘paving stones’. There are many grid and strip shadows under the pleura of both lungs |
| Patient 17 | The upper lung lobe, right lung middle lobe, right lung lower lobe, and left lower lobe of the lungs were scattered with multiple patchy ground glass density shadows, with the dorsal subpleural distribution in the lower lobe of the two lungs mainly accompanied by thickened lobular septa, and some of them were "paving stones" change |
| Patient 18 | The posterior segment of the right upper lobe, the posterior segment, the anterior segment of the right lung, the middle segment of the left lung, the posterior segment of the left lung, and the lower segment of the tongue are scattered in multiple patches of ground glass density shadows and some solid changes, and some lesions are accompanied by thickened lobular septa , Some of them changed like "paving stones". |
| Patient 19 | Interstitial pneumonia in both lungs, and some lesions are accompanied by thickened lobular septa , Some of them changed like "paving stones". |
| Patient 20 | The two lungs are scattered with multiple coronavirus pneumonia，The two lungs are scattered with multiple coronavirus pneumonia |
| Patient 21 | The two lungs are scattered with multiple coronavirus pneumonia，The two lungs are scattered with multiple coronavirus pneumonia |
| Patient 22 | The two lungs are scattered with multiple coronavirus pneumonia，The two lungs are scattered with multiple coronavirus pneumonia |
| Patient 23 | Two lungs are scattered in multiple patches of ground glass density shadows and some solid changes, and some lesions are accompanied by thickened lobular septa , Some of them changed like "paving stones". |
| Patient 24 | A large ground glass/sub-solid shadow can be seen on both lungs, considering the possibility of viral infection |
| Patient 25 | Multiple nodules and patches on both lungs, some mediastinal lymph nodes enlarged, a small amount of pleural effusion on both sides |
| Patient 26 | Multiple ground glass shadows on both lungs, pleural effusion on both sides, no thickening or adhesion on both sides of the pleura |
| Patient 27 | Multiple nodular foci in both lungs, a little pleural effusion on both sides |
| Patient 28 | Both lungs can be seen scattered glass/solid density patches and shadows, mostly under the pleura, and the local leaflet interval thickens, and multiple solid cords and strips are seen, and the bilateral pleura thickens and adheres |
| Patient 29 | Multiple ground glass and grid shadows can be seen in both lungs, no obvious enlargement of bilateral axillary and mediastinal lymph nodes, and no thickening or adhesion of bilateral pleura |
| Patient 30 | Right lung upper lobe, right lung middle lobe, right lung lower lobe, left lung lower lobe, left lower lobe, scattered plaque ground glass density shadow, some lesions with thickened lobular septa, some showed "paving stone"-like changes, and some were inflated Bronchial sign, thin-walled cystic lungless translucent shadow in both lungs |
| Patient 31 | Right upper lobe, right middle lobe, right lower lobe, left lower lobe, left lower lobe. Scattered plaque ground glass density shadow, some lesions with thickened lobular septa, and some changes like "paving stones". |
| Patient 32 | Coronary viral pneumonia in both lungs; scattered patchy patches in the upper lobe of the right lung and lower lobe of the left lung, with nodular consolidation and partial ground glass density shadows. Some lesions are accompanied by thickened leaflet interval, and some are changed in the shape of ‘paving stone’ |
| Patient 33 | The two lungs are scattered with multiple coronavirus pneumonia; the upper, middle and lower lobes of the right lung, and the left and upper lobes of the left lung are scattered with patchy ground glass density shadows. Some lesions are accompanied by thickened lobular septa, some are ‘paving stone’-like, and some have signs of inflatable bronchi |
| Patient 34 | Diffuse coronavirus pneumonia in both lungs; multiple spots of ground glass density in the right upper lobe, middle lobe, left upper lobe, and anterior segment of the lower lobe. Some lesions are accompanied by thickened leaflet intervals, and some are changed in the shape of ‘paving stones’. Local consolidation, blurred edges |
| Patient 35 | Diffuse coronavirus pneumonia in both lungs; the upper, middle and lower lobes of the right lung and the left and upper lobes of the left lung are scattered with patch-like ground glass density shadows. Some lesions are accompanied by thickened lobular septa, some are ‘paving stone’-like, and some have signs of inflatable bronchi |
| Patient 36 | Diffuse coronavirus pneumonia in both lungs; the upper, middle and lower lobes of the right lung, and the left and upper lobes of the left lung are scattered with patch-like ground glass density shadows, mainly dorsal subpleural distribution. With the thickening of the leaflet interval, part of it changes like ‘paving stone’, |
| Patient 37 | Right upper lobe, right middle lobe, right lower lobe, left upper lobe, left lower lobe, scattered patches of ground glass density shadow, some lesions with thickened lobular septa, some showed "paving stone"-like changes, and some were inflated Bronchial sign |
| Patient 38 | Diffuse coronavirus pneumonia in both lungs; right lung, left upper lobe, posterior segment of upper lobe, dorsal segment of lower lobe with multiple patch-like ground-glass density shadows, blurred borders, and local consolidation; no abnormal density shadows in the other two lungs |

**Supplementary Table 3. The pathogen distribution of the secondary infection patients.**

|  | **respiratory samples culture** | **respiratory samples mNGS (extra detected)** | **blood samples culture** | **blood samples mNGS (extra detected)** | **Urine samples** |
| --- | --- | --- | --- | --- | --- |
| **Patient 1** | *Acinetobacter baumannii;* |  |  | *HSV-2* |  |
| **Patient 2** | *Enterococcus faecium;Enterococcus faecalis;Aspergillus Fumigatus* | *HSV-1; Staphylococcus spp.* | *Staphylococcus haemolyticus；Candida albicans; Cryptococcus spp.* | *HSV-1* |  |
| **Patient 3** | *Staphylococcus spp.; Enterococcus faecium* | *Klebsiella pneumoniae; Acinetobacter baumannii* |  | *EBV* |  |
| **Patient 4** | *Acinetobacter baumannii;Enterococcus faecium* | *Klebsiella pneumoniae; Stenotrophomonas maltophilia* |  | *Candida albicans* |  |
| **Patient 5** |  | *HSV-1* | *Trichosporon asahii* |  |  |
| **Patient 6** | *Enterococcus faecium ;Klebsiella pneumoniae; Escherichia coli* |  | *Escherichia coli* |  | *Escherichia coli; Candida parapsilosis* |
| **Patient 7** | *Klebsiella pneumoniae; Chryseobacterium spp.;Acinetobacter baumannii* | *HSV-1; Enterococcus faecium* |  |  | *Acinetobacter baumannii* |
| **Patient 8** | *Enterococcus faecium; Penicillium spp.; Acinetobacter baumannii* | *Klebsiella pneumoniae; Mycobacterium intracellulare* |  |  |  |
| **Patient 9** | *Enterococcus faecium; Acinetobacter baumannii* | *HSV-1* | *Enterococcus faecium；Candida albicans* | *HSV-1* |  |
| **Patient 10** | *Enterococcus faecium;Klebsiella pneumoniae; Pseudomonas aeruginosa;Stenotrophomonas maltophilia* | *Mycoplasma hominis* | *Candida albicans* |  | *Candida albicans* |
| **Patient 11** | *Klebsiella pneumoniae; Enterococcus faecium* | *HSV-1* | *Candida albicans;Klebsiella pneumoniae* | | *Candida glabrata* |
| **Patient 12** |  | *HHV-6* | *Staphylococcus haemolyticus；* | *HHV-6* | *Candida albicans; Escherichia coli;  Klebsiella pneumoniae;  Pseudomonas aeruginosa* |
| **Patient 13** | *Aspergillus spp.* |  |  |  | *Escherichia coli* |
| **Patient 14** |  |  |  |  | *Enterococcus faecium* |
| **Patient 15** | *Staphylococcus spp* |  |  |  |  |
| **Patient 17** | *Klebsiella pneumoniae; Pseudomonas aeruginosa;Acinetobacter baumannii* |  |  |  |  |
| **Patient 18** | *Aspergillus spp.* |  |  |  |  |
| **Patient 20** | *Klebsiella pneumoniae* |  |  |  |  |
| **Patient 21** | *Klebsiella pneumoniae* |  |  |  |  |
| **Patient 22** | *Klebsiella pneumoniae* |  | *Klebsiella pneumoniae* |  |  |
| **Patient 23** | *Klebsiella pneumoniae* |  | *Klebsiella pneumoniae* |  |  |
| **Patient 24** | *Acinetobacter baumannii* |  | *Escherichia coli* |  |  |

**
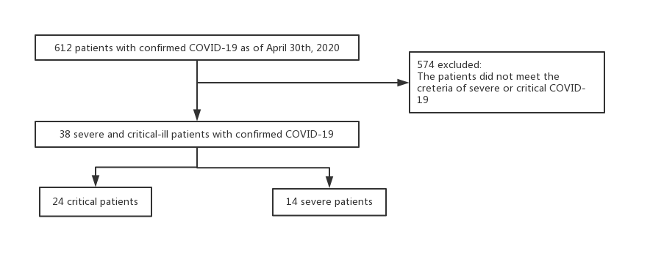
S****upplementary Figure. The flowchart of enrollment.**
