# Supplementary material for: Risks and features of secondary infections in severe and critical ill COVID-19 patients
Source: Emerg Microbes Infect. 2020 Sep 8;9(1):1958–64. doi: 10.1080/22221751.2020.1812437 (PMC8284966; doi:10.1080/22221751.2020.1812437)
Supplement: Revison_letter.docx [file TEMI_A_1812437_SM3313.docx]

TEMI-2020-0888

Title：Diagnosis strategy, risks and features of secondary infections in severe and critical ill COVID-19 patients

Prof. Shan Lu

Editor-in-Chief

Emerging Microbes & Infections

Prof. Shan Lu

Thanks for your letter and the reviewers’ comments about our manuscript (TEMI-2020-0888). The comments by the reviewers are constructive and helpful for improving the manuscript. We appreciate your interest of the subject and the chance you gave us to revise the manuscript. We also appreciate the general comments from the reviewer 1 “*Overall this is an important work with key data portrayed during a time where further information is required in various aspects of COVID-19.*” for your consideration for publication. We think that we have addressed reviewers’ comments to the best degree we could, and we hope this has met the reviewers’ and editor’s requests. Our detailed point-by-point responses to the comments are as follow:

***Responses to the Reviewer 1:***

***Question 1: Given the relatively large number of COVID-19 infections in the local hospital, this study only analysed 28 patients. It is important to add the flow of patient selection in the Method section.***

Thanks for the reviewer’s suggestion. We have added the flow chart of the patient selection in the result section of our article. Previously, we enrolled 378 patients admitted to Shanghai Public Health Clinical Center from 22^nd^ January to 21^st^ March to our study. We excluded the patients who did not meet the criteria of severe and critical criteria of COVID-19 according to the updated versions of the guidelines for the diagnosis and treatment of the disease issued by the National Health Commission of China. In the previous manuscript, 28 critical and severe COVID-19 patients were included for the analysis.

In order to improve the credibility of the article, we enrolled another 234 patients from Shanghai Public Health Clinical Center and Guanggu District, Tongji Hospital, Tongji Medical College, Huazhong University of Science and Technology from 22^nd^ January to April 30^th^ in the revised version. Finally, 24 critical-ill patients and 14 severe patients were included for the analysis.

***Question 2:*** ***Although invasive mechanical measures increase the risk of secondary infections, these measures are indispensable for severe and critical illness patients. What are the benefits of invasive mechanical measures? Authors should add the discussion.***

Thanks for the reviewer’s suggestion. Hypoxemia is common in hospitalized patients with COVID-19. The critical rate of COVID-19 in China is around 20%. In some hospitals in the United States, >25% of hospitalized patients require extra oxygenation or ventilation, mostly due to acute respiratory failure. Indirect evidence from other critical illnesses has suggested the oxygenation is important for severe pneumonia patients. The LOCO2 randomized controlled trial compared a conservative oxygen strategy (target SpO2 88% to 92%) to a liberal oxygen strategy (target SpO2 ≥96%) [1]. Mortality was increased by 8% and 14% among those who received the conservative oxygen therapy at Day 28 and Day 90. Previous guideline has demonstrated supportive care with mechanical ventilation is the cornerstone of patient management [2].

For the treatment of COVID-19, many guidelines, including guidelines issued by NIH and the National Health Commission of China guideline, has recommended the invasive mechanical measures as first priority in the treatment of critical COVID-19. Therefore, although the invasive mechanical measures increased the secondary infection rate of COVID-19 patients, it would decrease the overall mortality rate of the patients. We have added this part into the discussion.

*[1] Barrot L, Asfar P, Mauny F, et al. Liberal or conservative oxygen therapy for acute respiratory distress syndrome. N Engl J Med. 2020;382(11):999-1008.*

*[2] Fan E, Del Sorbo L, Goligher EC, et al. An Official American Thoracic Society/European Society of Intensive Care Medicine/Society of Critical Care Medicine Clinical Practice Guideline: Mechanical Ventilation in Adult Patients with Acute Respiratory Distress Syndrome [published correction appears in Am J Respir Crit Care Med. 2017 Jun 1;195(11):1540]. Am J Respir Crit Care Med. 2017;195(9):1253-1263. doi:10.1164/rccm.201703-0548ST*

***Responses to the Reviewer 2:***

***Question 1：The study sample size is relatively small, only 28 cases were included. However, the co-infection rate is relatively higher than other reports, the diagnosis of co-infection was not convincible based on their methods, and a settled conclusion could not be draw based on the results reported in the previous study. There are no clear explanations of the results.***

Thanks for the reviewer’s suggestion. In order to improve the credibility of the article, we increased the sample size in this revision. In the previous manuscript, we enrolled 378 patients admitted to Shanghai Public Health Clinical Center from 22^nd^ January to 21^st^ March, and we included 28 severe and critical patients for further analysis. In this revision, we enrolled another 234 patients from Shanghai Public Health Clinical Center and Guanggu District, Tongji Hospital, Tongji Medical College, Huazhong University of Science and Technology from 22nd January to April 30th. Finally, a total of 38 patients (24 critical patients and 14 severe patients) were included for further analysis.

Our study tends to analyze the secondary infection rate in critical-ill and severe patients, therefore, the overall secondary infection rate in our study might be a little higher than that in mild to moderate COVID-19 patients. What’s more, we believe the reason of a relatively higher secondary infection rate in our study might be due to the following reasons: patient characteristics (many of our patients are critically ill or in severe state), repeated culture of multiple samples, and extra detection of viruses and other pathogens through mNGS.

In order to make the study more convincible, we added more patients in the analysis of our study, and we further clarify the diagnosis criteria in this revision. Secondary infection was diagnosed when patients had clinical symptoms presenting infections or positive radiologic evidence, and a positive laboratory-confirmed etiologic result (culture positive or mNGS positive confirming by RT-PCR) after 48 hours of admission. And all We listed the co-infection pathogen results as followed:

|  | *respiratory samples culture* | *respiratory samples mNGS (extra detected)* | *blood samples culture* | *blood samples mNGS (extra detected)* | Urine samples |
| --- | --- | --- | --- | --- | --- |
| **Patient 1** | *Acinetobacter baumannii;* |  |  | *HSV-2* |  |
| **Patient 2** | *Enterococcus faecium;Enterococcus faecalis;Aspergillus Fumigatus* | *HSV-1; Staphylococcus spp.* | *Staphylococcus haemolyticus；Candida albicans; Cryptococcus spp.* | *HSV-1* |  |
| **Patient 3** | *Staphylococcus spp.; Enterococcus faecium* | *Klebsiella pneumoniae; Acinetobacter baumannii* |  | *EBV* |  |
| **Patient 4** | *Acinetobacter baumannii;Enterococcus faecium* | *Klebsiella pneumoniae; Stenotrophomonas maltophilia* |  | *Candida albicans* |  |
| **Patient 5** |  | *HSV-1* | *Trichosporon asahii* |  |  |
| **Patient 6** | *Enterococcus faecium; Klebsiella pneumoniae; Escherichia coli* |  | *Escherichia coli* |  | Escherichia coli; Candida parapsilosis |
| **Patient 7** | *Klebsiella pneumoniae; Chryseobacterium spp.; Acinetobacter baumannii* | *HSV-1; Enterococcus faecium* |  |  | Acinetobacter baumannii |
| **Patient 8** | *Enterococcus faecium; Penicillium spp.; Acinetobacter baumannii* | *Klebsiella pneumoniae; Mycobacterium intracellulare* |  |  |  |
| **Patient 9** | *Enterococcus faecium; Acinetobacter baumannii* | *HSV-1* | *Enterococcus faecium；Candida albicans* | *HSV-1* |  |
| **Patient 10** | *Enterococcus faecium;Klebsiella pneumoniae; Pseudomonas aeruginosa;Stenotrophomonas maltophilia* | *Mycoplasma hominis* | *Candida albicans* |  | Candida albicans |
| **Patient 11** | *Klebsiella pneumoniae; Enterococcus faecium* | *HSV-1* | *Candida albicans;Klebsiella pneumoniae* | | *Candida glabrata* |
| **Patient 12** |  | *HHV-6* | *Staphylococcus haemolyticus；* | *HHV-6* | *Candida albicans; Escherichia coli;  Klebsiella pneumoniae;  Pseudomonas aeruginosa* |
| **Patient 13** | *Aspergillus spp.* |  |  |  | *Escherichia coli* |
| **Patient 14** |  |  |  |  | *Enterococcus faecium* |
| **Patient 15** | *Staphylococcus spp* |  |  |  |  |
| **Patient 17** | *Klebsiella pneumoniae; Pseudomonas aeruginosa;Acinetobacter baumannii* |  |  |  |  |
| **Patient 18** | *Aspergillus spp.* |  |  |  |  |
| **Patient 20** | *Klebsiella pneumoniae* |  |  |  |  |
| **Patient 21** | *Klebsiella pneumoniae* |  |  |  |  |
| **Patient 22** | *Klebsiella pneumoniae* |  | *Klebsiella pneumoniae* |  |  |
| **Patient 23** | *Klebsiella pneumoniae* |  | *Klebsiella pneumoniae* |  |  |
| **Patient 24** | *Acinetobacter baumannii* |  | *Escherichia coli* |  |  |

***Question 2: Did you evaluate the usefulness of dynamic (daily) PCT monitoring in your case series as a predictor of bacterial co-infection?***

Thanks for the reviewer’s question. We have recorded the dynamic PCT level of our cases series. In order to investigate PCT predictable value of COVID-19 bacterial co-infection, we chose 5 time points listed as followed: the day patients admitted to the hospital (Baseline), the first culture positive day (Day 0), and the 3 days before culture positive day (Day -1, Day -2, Day -3). The median PCT baseline level of severe and critical COVID-19 patients was 0.46 ng/ml, which is higher than the normal stage 0.05ng/ml. However, we found the median PCT level on the culture positive day and 1-2 days before the culture positive day was significantly higher than the baseline (1.432 vs 0.459, *P=0.008*; 0.875 vs 0.459, *P*=0.018; 1.092 vs 0.459, *P*=0.031). The results demonstrated the dynamic PCT may serve as a predictor of bacterial co-infection.

***Question 3: Not all the patients got routine mNGS for monitoring of the co-infection. When do you considered it’s ‘necessary’ for a mNGS test? What is the criteria for differentiating a colonization or co-infection with a positive mNGS in this study? Usually several organisms maybe found in the mNGS, how do you decide which one(or ones) were the responsible organisms for the co-infection?***

Thanks for the reviewer’s question. All the patients met the criteria of critical COVID-19 were considered to get mNGS test of blood and sputum samples when they were suspicious for secondary infection (when they had clinical symptoms presenting infections or positive radiologic evidence) 48 hours after admission.

A criteria for a positive mNGS result interpretation was referred to the criteria that has been published in the Clinical Infectious Disease[1]. Furthermore, we had confirmed the extra mNGS results by RT-PCR if the causative pathogens interpreted by mNGS criteria were not culture positive or routine PCR positive.

However, whether the identified pathogen responsible for the co-infection or colonization was decided by many conditions including the species of the pathogens, pathopoiesis of the pathogens, the immune-status of the patients, clinical assessment of the physicians, and etc. For example, it is hard to decide whether pathogens like *Acinetobacter baumannii* is pathogenic when the culture or PCR was positive in some clinical circumstances.

The judgement of whether causative agents was co-infection or colonization was therefore largely depended on the clinical physicians’ advise when we were faced with such kinds of results. In the revised version, we have reanalyzed the causative pathogens with the clinical physician expert groups and made the final diagnosis of causative agents according to the experts’ discussion results. We listed the pathogen results in the answer of Question 1.

*[1] Miao Q, Ma Y, Wang Q, et al. Microbiological Diagnostic Performance of Metagenomic Next-generation Sequencing When Applied to Clinical Practice. Clin Infect Dis. 2018;67(suppl_2):S231-S240. doi:10.1093/cid/ciy693*

***Question 4:*** ***The source for bloodstream co-infection was not clarified. Was it related to the central venous catheters or associated to respiratory infections related sepsis?***

Thank you for your suggestions. We clarified the source of pathogens in blood-stream infections according to discussion results of the clinical experts’ groups. They made the final diagnosis according to the evaluation of clinical characteristics, laboratory results, culture and mNGS results. We listed results in the following table:

|  | Sources of blood-stream secondary infection |
| --- | --- |
| Patient 1 | *HSV-2:* opportunistic infection (urinary tract) |
| Patient 2 | HSV-1: respiratory infection (respiratory mNGS: HSV-1) *Staphylococcus haemolyticus:* intravascular device (from IVC or CRRT)  *Candida albicans:* opportunistic infection (respiratory tract, or urinary tract)  *Cryptococcus spp.:* opportunistic infection |
| Patient 3 | EBV: opportunistic infection |
| Patient 4 | *Candida albicans:* opportunistic infection (respiratory tract, or urinary tract) |
| Patient 5 | *Trichosporon asahii:* intravascular device (IVC culture: *Trichosporon asahii*) |
| Patient 6 | *Escherichia coli*：urinary infection (urinary culture: *Escherichia coli*) |
| Patient 9 | *Enterococcus faecium:* respiratory infection (respiratory sample culture: *Enterococcus faecium*)  *Candida albicans:* opportunistic infection (respiratory tract, or urinary tract) *HSV-1:* respiratory infection (respiratory mNGS: HSV-1) |
| Patient 10 | *Candida albicans*: opportunistic infection (respiratory tract, or urinary tract) |
| Patient 11 | *Candida albicans:* opportunistic infection (respiratory tract, or urinary tract) *Klebsiella pneumoniae:* respiratory infection (respiratory sample culture: *Klebsiella pneumoniae*) |
| Patient 12 | *HHV-6*: respiratory infection (respiratory mNGS: HHV-6) |
| Patient 22 | *Klebsiella pneumoniae*: respiratory infection (respiratory sample culture: *Klebsiella pneumoniae*) |
| Patient 23 | *Klebsiella pneumoniae*: respiratory infection (respiratory sample culture: *Klebsiella pneumoniae*) |
| Patient 24 | *Escherichia coli*: respiratory infection (respiratory sample culture: *Escherichia coli*) |

***Question 5: Co-infection with HSV, EBV or CMV usually needs pathological evidence, did any patients get biopsy in this study? How the authors made the diagnosis of viral co-infection in this cases other than positive mNGS ?***

Thanks for the reviewer’s question. We did the routine blood EBV and CMV PT-PCR for every severe and critical patient. The results of the mNGS were confirmed by the routine blood EBV and CMV RT-PCR results. For the patients with HSV or respiratory CMV/ EBV infection, we had confirmed the extra mNGS results by RT-PCR if the causative pathogens interpreted by mNGS criteria were not culture positive or routine PCR positive. The diagnosis of the specific pathogen infection was made by the clinical physician groups according to the discussion results.

***Question 6： Candida spp found in respiratory and urinary samples should not be appropriate to treated as an evidence for a co-infection!***
Thank you for your suggestions. We have carefully checked the Candida spp. in our list , and reanalyzed the causative pathogens with the clinical physician expert groups and made the final diagnosis of pathogens according to the experts’ discussion results. In respiratory samples, we detected one *Candida parapsilosis* and one *Candida tropicali*s. As suggested by your suggestions and previous criteria to find true pathogens [1], we have removed the Candida spp. in respiratory samples.

In urinary samples, we reported *Candida albicans, Candida parapsilosis* and *Candida glabrata* infections. However, repeated urinary infection culture of these patients has showed high-level *Candida spp.* positive results for several time in a period of time. Previous study showed high-level of candiduria may be associated with a concurrent blood culture submission, which will lead to poor progonsis [2] and suggested systemic antifungal treatment in critically ill patients [3]. In our study, the clinical physician expert groups considered these patients had secondary urinary infection of *Candida spp* and should be treated.

*[1] Miao Q, Ma Y, Wang Q, et al. Microbiological Diagnostic Performance of Metagenomic Next-generation Sequencing When Applied to Clinical Practice. Clinical Infectious Diseases. 2018;67(suppl_2): S231-S240.*

*[2] Huang Susan S,Septimus Edward,Hayden Mary K et al. Effect of body surface decolonisation on bacteriuria and candiduria in intensive care units: an analysis of a cluster-randomised trial.[J] .Lancet Infect Dis, 2016, 16: 70-79.*

*[3] Hope William W,Assessing candiduria in a critically ill patient.[J] .BMJ, 2009, 338: b2289.*

***Question 7：* The relationship of catheters inserted and its related organism isolation was not clearly clarified by the data in results section.**

Thank you for your suggestions. We have carried out analyses of sources of blood-stream infections. A total of 25, 8 and 10 patients received IVC, ECMO and CRRT respectively and 20 patients accepted urinary catheters. 13 patients only received IVC, 4 patients both received IVC and CRRT, 2 patients both received IVC and ECMO. 6 patients received IVC, CRRT and ECMO.

We have added analyses of detected blood-stream infection pathogens following invasive operations in Table 2B. As mentioned in the question 4, we clarified the source of pathogens in blood-stream infections as following:

|  | Sources of blood-stream secondary infection |
| --- | --- |
| Patient 1 | *HSV-2:* opportunistic infection (urinary tract) |
| Patient 2 | HSV-1: respiratory infection (respiratory mNGS: HSV-1) *Staphylococcus haemolyticus:* intravascular device (from IVC or CRRT)  *Candida albicans:* opportunistic infection (respiratory tract, or urinary tract)  *Cryptococcus spp.:* opportunistic infection |
| Patient 3 | EBV: opportunistic infection |
| Patient 4 | *Candida albicans:* opportunistic infection (respiratory tract, or urinary tract) |
| Patient 5 | *Trichosporon asahii:* intravascular device (IVC cultue: *Trichosporon asahii*) |
| Patient 6 | *Escherichia coli*：urinary infection (urinary culture: *Escherichia coli*) |
| Patient 9 | *Enterococcus faecium:* respiratory infection (respiratory sample culture: *Enterococcus faecium*)  *Candida albicans:* opportunistic infection (respiratory tract, or urinary tract) *HSV-1:* respiratory infection (respiratory mNGS: HSV-1) |
| Patient 10 | *Candida albicans*: opportunistic infection (respiratory tract, or urinary tract) |
| Patient 11 | *Candida albicans:* opportunistic infection (respiratory tract, or urinary tract) *Klebsiella pneumoniae:* respiratory infection (respiratory sample culture: *Klebsiella pneumoniae*) |
| Patient 12 | *HHV-6*: respiratory infection (respiratory mNGS: HHV-6) |
| Patient 22 | *Klebsiella pneumoniae*: respiratory infection (respiratory sample culture: *Klebsiella pneumoniae*) |
| Patient 23 | *Klebsiella pneumoniae*: respiratory infection (respiratory sample culture: *Klebsiella pneumoniae*) |
| Patient 24 | *Escherichia coli*: respiratory infection (respiratory sample culture: *Escherichia coli*) |

***Question 8： The data about different sites of pathogen isolation and its relationship with different support therapy and catheters used should be added in the results. Such as how many patients received CRRT? How many patients received IVC and urinary catheter insertion?***

Thank you for your suggestions. We have added data of CRRT (continuous renal replacement therapy) use, catheters use. A total of 25, 8 and 10 patients received IVC, ECMO and CRRT respectively and 20 patients accepted urinary catheters.

We next performed analyses of compositions of blood-stream infection pathogens and differences in median occurrence time after invasive operations. As shown in Table 2b, the median time of infection after IVC (intravenous catheter), ECMO and CRRT (continuous renal replacement therapy) was 12, 12 and 10 days respectively. A total of 19 pathogens were detected after IVC, of which 5 pathogens were gram-negative bacteria, 3 were gram-positive bacteria, 4 were viruses and 7 were fungi. Fungi accounted for 44.44% (4/9) and 40.00% (4/10) after ECMO and CRRT operations separately (Table 2b and Figure 2B). We have added the context into the revised version.

|  | IVC | ECMO | CRRT |
| --- | --- | --- | --- |
| Cases (n) | 25 | 8 | 10 |
| Perform time (d, mean ± SD) | 8.76±6.55 | 14.88±9.20 | 19.2±14.28 |
| Proportion of infection (%) | 52.00% (13/25) | 75.00% (6/8) | 60.00% (6/10) |
| Occurrence time of infection in patients (median(range)) | 12(1-35) | 12(1-21) | 10.5(2-23) |
| Detected respiratory pathogens (n) | 19 | 9 | 10 |

***Question 9: How the use of mNGS change your clinical practice or drug therapy, then the outcome? Please clarify.***

Thanks for the reviewer’s question. According to the mNGS results of respiratory and blood samples, 6 (25.00%) of the patients changed the drug therapy. We prescribed extra antibiotics and antiviral drugs to those patients. Finally, in 24 critical patients who had mNGS test, the overall 60-day mortality rate is 37.5%, and the final morality rate is 50.00%. We have added the context into the discussion.

***Question 10：The discussion is too long and it should be more related to the data from your own results and the relationship between the cause of infection and the types of co-infection, and the mechanism between the co-infection and the outcome.***

Thanks for the reviewer’s question. We have rewritten the discussion in the manuscript.

***Question 11: Did you use any antibiotics to prevent the co-infection before the pathogen isolation? Or what was your anti-infection strategy when patients admitted to the hospital and ICU? Does it*** ***associated with the co-infection or the antimicrobial susceptiveness of the isolated pathogens?***

Thanks for the reviewer’s question. To prevent the increase of the antimicrobial resistance rate. We did not generally use antibiotic to prevent secondary infection in COVID-19 patients. However, we prescribed 4 patients with preventive antibiotic therapy of moxifloxacin because they received methylprednisolone of more than 0.4mg/kg/day for over 5 days before any culture positive results. Once the culture results reported positive, we would change their antibiotics according to the drug sensitivity.

Among 4 patients receiving preventive moxifloxacin treatment, only one patient later had a culture positive for *Acinetobacter baumannii* that is quinolone natural resistance. Therefore, we believe the preventive therapy might had a limited impact on the co-infection or the antimicrobial resistance of the isolated pathogens.

***Minor***

***Question 1: Page 7 line 111> PaO2/FiO2 should be “******PaO2/FiO2 ratio or*** ***PFR”, it should not be called as “oxygenation index”, PFR and OI are two different concepts.***

Thanks for the reviewer’s suggestion. We may use ***PaO2/FiO2 ratio*** instead of oxygenation index in our article.

***Question 2: Page 8 line 118> Why written consents were not obtained from the patients or their next of kins?***

Thanks for the reviewer’s suggestion. We have obtained some proportions of the written consents of the enrolled patients first. However, few patients were in critical-ill status when they transferred to the hospitals, and their family member did not come with them together. We obtained oral consent from these patients’ members first at that time. We have obtained the written consents of all the patients or their family members retrospectively.

***Question 3: Page 9 line 145> reads “Total of 28 severe and critical…” should be “A total of 28…”***

Thank you for your suggestions. We have made revision and changed the sentence to ‘A total of 28 severe and critical COVID-19 patients were enrolled.’

***Question 4: Page 10 line 151> reads “were different between secondary infection and non-infection patients”, I am not agree with calling the patients who didn’t get secondary infection as “non-infection patients”, they were infected with COVID actually.***

Thank you for your suggestions. We are sorry for the miss use of ‘non-infection’ word, we have now made revisions and changed them to ‘non-secondary infections’.

***Question 5: Supplementary Table, row 2> it is hard to understand why only one case of 10 was severe of in critical illness? Should all the patients included in this study be severe cases?***
Thank you for your suggestions. We are sorry for the wrong title of row 2 in Supplementary Table. It should be ‘Critical status’. We here analyzed the numbers of critical patients in secondary -infections and non-secondary infections respectively. And it illustrated that there were 20 and 2 critical patients in secondary infection and non-secondary infection group respectively.

***Responses to the Reviewer 3:***

***MAY REMOVE*** ***DIAGNOSIS STRATEGY AS THE FOCUS IS ON RISK AND FEATURES OF SECOBDARY INFECTIONS***

Thanks for the reviewer’s suggestion. We will remove the ***DIAGNOSIS STRATEGY*** from the title of our article.

***INTRODUCTION***

***Can you just use secondary infection and not shift to*** ***superinfection or nosocomial infection in the whole manuscript?***

Thanks for the reviewer’s suggestion. We have changed the use of superinfection or nosocomial infection into the secondary infection in the whole manuscript.

***METHODS***

***As this is a retrospective study, “enrolled” should rather be INCLUDED (line 104)***

Thanks for the reviewer’s suggestion. We have changed the “enrolled” into “included”

***Inclusion criteria “severe and critically ill COVID 19” patients – were these discharge diagnoses as this study was retrospective.***

Thanks for the reviewer’s suggestion. The diagnosis of our study is the discharge diagnosis.

***Sample procedures – “We collect”: since again, this is retrospective, it should be stated as: Samples collected from patients included.... (line 121). The assumption here is that all patients had these samples collected from them.***

Thanks for the reviewer’s suggestion. We have changed the “we collect” into “Samples collected from patients included....”.

***Please define your terms,*** ***invasive mechanical ventilation, ECMO.***

Thanks for the reviewer’s suggestion. invasive mechanical ventilation includes tracheal intubation and tracheotomy in the article. ECMO was defined as extra-corporeal membrane oxygenation

***What was the criteria for doing mNGS? (line 131) How many had samples, from what site (respiratory, blood, urine) were subjected to mNGS?***

Thanks for the reviewer’s question. All the patients met the criteria of critical-ill COVID-19 were considered to get mNGS test of blood and sputum samples when they were suspicious for secondary infection (when they had clinical symptoms presenting infections or positive radiologic evidence) 48 hours after admission.

***RESULTS***

***It would be clearer if you described the 28 cases on admission as to the management***

***started: how many were on HFNC at the start? How many had tracheal intubation?*** ***Tracheostomy? Then eventually during the patients’ course, how many*** ***were intubated or put on ECMO?***

Thanks for the reviewer’s question. In the 38 patients, 29 patients were on HFNC, and 9 patients had tracheal intubation, and none of the patients was tracheostomy at the start. And during the patients’ course, 23 patients and 8 patients were and put on ECMO, respectively.

***Please include chest x-ray or chest CT findings for both groups (in the text and in the table).***

Thanks for the reviewer’s suggestion. We have added the chest x-ray or chest CT findings for both groups in the text and in the supplementary table. We have listed the CT or X-ray findings below:

|  | CT or X-ray findings |
| --- | --- |
| Patient 1 | Right lower lobe, upper left lobe, left lower lobe showed scattered patch-like ground glass density shadows, some lesions were accompanied by thickened lobular septa, some showed "paving stone"-like changes and bronchial inflation Sign |
| Patient 2 | flakes and large ground glass-like density increase were seen in the field of the two lungs, |
| Patient 3 | The upper lobe of the right lung, the middle lobe, the lower lobe of the right lung, and the upper and lower lobes， some lesions were accompanied by thickened lobular septa, some showed "paving stone"-like changes and bronchial inflation Sign |
| Patient 4 | Right upper lobe， Right lower lobe， Left lower lobe，and Left upper lobesome lesions were accompanied by thickened lobular septa, some showed "paving stone"-like changes and bronchial inflation Sign. |
| Patient 5 | The upper, middle and lower lobes of the right lung and the upper and lower lobes of the left lung are scattered with patchy ground glass density shadows, mainly distributed in the dorsal subpleural area, with the thickened leaflet interval in the area, and some of them are changed as "paving stones" |
| Patient 6 | There were multiple patchy ground glass density shadows with partial consolidation under the upper pleura of the two lungs, and a few patchy ground glass density shadows in the right lung midfield. In the lower lobe of the lungs, there were multiple lamellar consolidations and fibrous cord shadows, in which air bronchial signs were seen; the left lower lobe bronchus was slightly dilated, and there was no obvious abnormality in the bronchial wall. |
| Patient 7 | In the upper and lower lobes of the right lung, and the upper and lower lobes of the left lung, scattered patch-like ground glass density shadows were seen. Some lesions are accompanied by thickened lobular interval, some are changed in the shape of ‘paving stone’, the boundary is not clear, and some see signs of inflatable bronchi |
| Patient 8 | The right upper lobe, the right middle lobe, the right lower lobe, and the left upper lobe and the left lower lobe showed scattered patch-like ground glass density shadows. Some lesions were thickened with lobular septa,with inflatable Bronchial Sign |
| Patient 9 | Coronary virus pneumonia was diffuse in both lungs, and there were diffuse patchy, ground glass-like exudative solid changes in both lungs, and the border was unclear. |
| Patient 10 | The upper, middle and lower lobes of the right lung and the upper and lower lobes of the left lung were scattered with patch-like ground glass density shadows. Some lesions are accompanied by thickened lobular septa, some are ‘paving stone’-like, and some have airy bronchial signs. Pleural effusions on both sides |
| Patient 11 | A small amount of pleural effusion in the pleural cavity; the upper, middle and lower lobes of the right lung, and the scattered glazed glass density shadows in the upper and lower lobes of the left lung. Some lesions are accompanied by thickened lobular septa, some are ‘paving stone’-like, and some have signs of inflatable bronchi |
| Patient 12 | Diffuse coronavirus pneumonia in both lungs with focal fibrosis, and a small amount of fluid in the left pleural cavity. The right upper lobe, middle lobe, right lower lobe, left and upper lobe of the left lung are scattered in patchy ground glass shadows, some lesions are thickened with lobular septa, some are "paving stone" changes, and some have inflatable bronchi |
| Patient 13 | Right upper lobe, right middle lobe, right lower lobe, left lower lobe, left lower lobe, left lower lobe, scattered patchy ground glass density shadow, some lesions with thickened lobular septa, some showed "paving stone"-like changes, and some were inflated Bronchial sign |
| Patient 14 | Right upper lobe, right middle lobe, right lower lobe, left lower lobe, left lower lobe, left lower lobe, scattered patchy ground glass density shadow, some lesions with thickened lobular septa, some showed "paving stone"-like changes, and some were inflated Bronchial sign |
| Patient 15 | Right upper lobe of right lung Middle lobe of right lung Lower lobe of right lung Left lobe of left lung See scattered patch of ground glass density shadow, some lesions with thickened lobular septa, some appear "paving stone"-like changes, some see inflation bronchial signs |
| Patient 16 | Coronary viral pneumonia in both lungs; patchy ground-glass density scattered scattered under the pleura of both lungs. Some lesions are accompanied by thickened lobular septa, and some are changed in the shape of ‘paving stones’. There are many grid and strip shadows under the pleura of both lungs |
| Patient 17 | The upper lung lobe, right lung middle lobe, right lung lower lobe, and left lower lobe of the lungs were scattered with multiple patchy ground glass density shadows, with the dorsal subpleural distribution in the lower lobe of the two lungs mainly accompanied by thickened lobular septa, and some of them were "paving stones" change |
| Patient 18 | The posterior segment of the right upper lobe, the posterior segment, the anterior segment of the right lung, the middle segment of the left lung, the posterior segment of the left lung, and the lower segment of the tongue are scattered in multiple patches of ground glass density shadows and some solid changes, and some lesions are accompanied by thickened lobular septa , Some of them changed like "paving stones". |
| Patient 19 | Interstitial pneumonia in both lungs, and some lesions are accompanied by thickened lobular septa , Some of them changed like "paving stones". |
| Patient 20 | The two lungs are scattered with multiple coronavirus pneumonia，The two lungs are scattered with multiple coronavirus pneumonia |
| Patient 21 | The two lungs are scattered with multiple coronavirus pneumonia，The two lungs are scattered with multiple coronavirus pneumonia |
| Patient 22 | The two lungs are scattered with multiple coronavirus pneumonia，The two lungs are scattered with multiple coronavirus pneumonia |
| Patient 23 | Two lungs are scattered in multiple patches of ground glass density shadows and some solid changes, and some lesions are accompanied by thickened lobular septa , Some of them changed like "paving stones". |
| Patient 24 | A large ground glass/sub-solid shadow can be seen on both lungs, considering the possibility of viral infection |
| Patient 25 | Multiple nodules and patches on both lungs, some mediastinal lymph nodes enlarged, a small amount of pleural effusion on both sides |
| Patient 26 | Multiple ground glass shadows on both lungs, pleural effusion on both sides, no thickening or adhesion on both sides of the pleura |
| Patient 27 | Multiple nodular foci in both lungs, a little pleural effusion on both sides |
| Patient 28 | Both lungs can be seen scattered glass/solid density patches and shadows, mostly under the pleura, and the local leaflet interval thickens, and multiple solid cords and strips are seen, and the bilateral pleura thickens and adheres |
| Patient 29 | Multiple ground glass and grid shadows can be seen in both lungs, no obvious enlargement of bilateral axillary and mediastinal lymph nodes, and no thickening or adhesion of bilateral pleura |
| Patient 30 | Right lung upper lobe, right lung middle lobe, right lung lower lobe, left lung lower lobe, left lower lobe, scattered plaque ground glass density shadow, some lesions with thickened lobular septa, some showed "paving stone"-like changes, and some were inflated Bronchial sign, thin-walled cystic lungless translucent shadow in both lungs |
| Patient 31 | Right upper lobe, right middle lobe, right lower lobe, left lower lobe, left lower lobe. Scattered plaque ground glass density shadow, some lesions with thickened lobular septa, and some changes like "paving stones". |
| Patient 32 | Coronary viral pneumonia in both lungs; scattered patchy patches in the upper lobe of the right lung and lower lobe of the left lung, with nodular consolidation and partial ground glass density shadows. Some lesions are accompanied by thickened leaflet interval, and some are changed in the shape of ‘paving stone’ |
| Patient 33 | The two lungs are scattered with multiple coronavirus pneumonia; the upper, middle and lower lobes of the right lung, and the left and upper lobes of the left lung are scattered with patchy ground glass density shadows. Some lesions are accompanied by thickened lobular septa, some are ‘paving stone’-like, and some have signs of inflatable bronchi |
| Patient 34 | Diffuse coronavirus pneumonia in both lungs; multiple spots of ground glass density in the right upper lobe, middle lobe, left upper lobe, and anterior segment of the lower lobe. Some lesions are accompanied by thickened leaflet intervals, and some are changed in the shape of ‘paving stones’. Local consolidation, blurred edges |
| Patient 35 | Diffuse coronavirus pneumonia in both lungs; the upper, middle and lower lobes of the right lung and the left and upper lobes of the left lung are scattered with patch-like ground glass density shadows. Some lesions are accompanied by thickened lobular septa, some are ‘paving stone’-like, and some have signs of inflatable bronchi |
| Patient 36 | Diffuse coronavirus pneumonia in both lungs; the upper, middle and lower lobes of the right lung, and the left and upper lobes of the left lung are scattered with patch-like ground glass density shadows, mainly dorsal subpleural distribution. With the thickening of the leaflet interval, part of it changes like ‘paving stone’, |
| Patient 37 | Right upper lobe, right middle lobe, right lower lobe, left upper lobe, left lower lobe, scattered patches of ground glass density shadow, some lesions with thickened lobular septa, some showed "paving stone"-like changes, and some were inflated Bronchial sign |
| Patient 38 | Diffuse coronavirus pneumonia in both lungs; right lung, left upper lobe, posterior segment of upper lobe, dorsal segment of lower lobe with multiple patch-like ground-glass density shadows, blurred borders, and local consolidation; no abnormal density shadows in the other two lungs |

***I would re write this:*** ***overall,18 (64.3%) developed secondary infection. Secondary***

***infection rate in the critically ill patients was much higher than that in severe patients [94.1% (16/17) vs 18.2% (2/11), p<0.001]. The second sentence may not be necessary as you have established this difference already.***

Thanks for the reviewer’s suggestion. We have rewrote the sentence as “overall,22 (57.89%) developed secondary infection. Secondary-infection rate in critical patients is much higher than that in severe patients (83.33% (20/24) vs 14.29% (2/14), P<0.0001).”

***Please report only the true respiratory pathogens (as not all those detected***

***from the respiratory tract are clinically significant. Refer to Winn et al. Koneman’s Color Atlas and Textbook of Diagnostic Microbiology. 2006). This should be for the*** ***whole manuscript and the Figure 1.***

***by mNGS, how many and what pathogens were detected from the respiratory tract and blood, among severe and critically ill COVID patints?***

Thanks for the reviewer’s suggestion. We have reconsidered the respiratory pathogens referred to Winn et al. Koneman’s Color Atlas and Textbook of Diagnostic Microbiology. 2006) , and reanalyzed the causative pathogens with the clinical physician groups and made the final diagnosis of causative agents according to the discussion results.

We listed the mNGS and culture results as followed and renewed the data whole manuscript and the Figure 1.

|  | **respiratory samples culture** | **respiratory samples mNGS (extra detected)** | **blood samples culture** | **blood samples mNGS (extra detected)** | **Urine samples** |
| --- | --- | --- | --- | --- | --- |
| **Patient 1** | *Acinetobacter baumannii;* |  |  | *HSV-2* |  |
| **Patient 2** | *Enterococcus faecium;Enterococcus faecalis;Aspergillus Fumigatus* | *HSV-1; Staphylococcus spp.* | *Staphylococcus haemolyticus；Candida albicans; Cryptococcus spp.* | *HSV-1* |  |
| **Patient 3** | *Staphylococcus spp.; Enterococcus faecium* | *Klebsiella pneumoniae; Acinetobacter baumannii* |  | *EBV* |  |
| **Patient 4** | *Acinetobacter baumannii;Enterococcus faecium* | *Klebsiella pneumoniae; Stenotrophomonas maltophilia* |  | *Candida albicans* |  |
| **Patient 5** |  | *HSV-1* | *Trichosporon asahii* |  |  |
| **Patient 6** | *Enterococcus faecium ;Klebsiella pneumoniae; Escherichia coli* |  | *Escherichia coli* |  | *Escherichia coli; Candida parapsilosis* |
| **Patient 7** | *Klebsiella pneumoniae; Chryseobacterium spp.;Acinetobacter baumannii* | *HSV-1; Enterococcus faecium* |  |  | *Acinetobacter baumannii* |
| **Patient 8** | *Enterococcus faecium; Penicillium spp.; Acinetobacter baumannii* | *Klebsiella pneumoniae; Mycobacterium intracellulare* |  |  |  |
| **Patient 9** | *Enterococcus faecium; Acinetobacter baumannii* | *HSV-1* | *Enterococcus faecium；Candida albicans* | *HSV-1* |  |
| **Patient 10** | *Enterococcus faecium;Klebsiella pneumoniae; Pseudomonas aeruginosa;Stenotrophomonas maltophilia* | *Mycoplasma hominis* | *Candida albicans* |  | *Candida albicans* |
| **Patient 11** | *Klebsiella pneumoniae; Enterococcus faecium* | *HSV-1* | *Candida albicans;Klebsiella pneumoniae* | | *Candida glabrata* |
| **Patient 12** |  | *HHV-6* | *Staphylococcus haemolyticus；* | *HHV-6* | *Candida albicans; Escherichia coli;  Klebsiella pneumoniae;  Pseudomonas aeruginosa* |
| **Patient 13** | *Aspergillus spp.* |  |  |  | *Escherichia coli* |
| **Patient 14** |  |  |  |  | *Enterococcus faecium* |
| **Patient 15** | *Staphylococcus spp* |  |  |  |  |
| **Patient 17** | *Klebsiella pneumoniae; Pseudomonas aeruginosa;Acinetobacter baumannii* |  |  |  |  |
| **Patient 18** | *Aspergillus spp.* |  |  |  |  |
| **Patient 20** | *Klebsiella pneumoniae* |  |  |  |  |
| **Patient 21** | *Klebsiella pneumoniae* |  |  |  |  |
| **Patient 22** | *Klebsiella pneumoniae* |  | *Klebsiella pneumoniae* |  |  |
| **Patient 23** | *Klebsiella pneumoniae* |  | *Klebsiella pneumoniae* |  |  |
| **Patient 24** | *Acinetobacter baumannii* |  | *Escherichia coli* |  |  |

***What*** ***specific pathogens were detected from what site using the different mechanical***

***ventilation support and ECMO? I would assume that blood borne pathogens were more***

***common in ECMO.***

Thanks for the reviewer’s suggestion. In our study, 23 patients received invasive mechanical ventilation, 8 of them received the ECMO therapy. We have listed the detected pathogens after the invasive procedure in the following table. We found 75.00%(6/8) of the patients receiving ECMO therapy and 46.67%(7/15) of the patients who only received the invasive mechanical ventilation developed blood-stream infection, respectively. We listed the possible source of the blood borne pathogens in the following table.

|  | Respiratory infection pathogens | Blood-stream infection pathogens |
| --- | --- | --- |
| Invasive mechanical ventilation | Acinetobacter baumannii;  Enterococcus faecium;  Staphylococcus spp.  Klebsiella pneumoniae;  HSV-1;  Escherichia coli；  Chryseobacterium spp.  *HHV-6* | *HHV-6*  *EBV*  *Candida albicans*  Klebsiella pneumoniae  Staphylococcus spp.  *Trichosporon asahii*  *Escherichia coli* |
| Invasive mechanical ventilation and ECMO | Acinetobacter baumannii;  Enterococcus faecium;  Enterococcus faecalis;  Aspergillus spp.；  HSV-1;  Staphylococcus spp.  Klebsiella pneumoniae;  Stenotrophomonas maltophilia；  Penicillium spp.;  Mycobacterium intracellulare;  *Mycoplasma hominis*  Pseudomonas aeruginosa | *HSV-1*  *HSV-2*  *Candida albicans*  Cryptococcus spp.  Enterococcus faecium  Klebsiella pneumoniae |

|  | Sources of blood-stream secondary infection | Invasive procedure |
| --- | --- | --- |
| Patient 1 | *HSV-2:* opportunistic infection (urinary tract) | *ECMO+* invasive mechanical ventilation |
| Patient 2 | HSV-1: respiratory infection (respiratory mNGS: HSV-1) *Staphylococcus haemolyticus:* intravascular device (from IVC or CRRT)  *Candida albicans:* opportunistic infection (respiratory tract, or urinary tract)  *Cryptococcus spp.:* opportunistic infection | *ECMO+* invasive mechanical ventilation |
| Patient 3 | EBV: opportunistic infection | invasive mechanical ventilation |
| Patient 4 | *Candida albicans:* opportunistic infection (respiratory tract, or urinary tract) | *ECMO+* invasive mechanical ventilation |
| Patient 5 | *Trichosporon asahii:* intravascular device (IVC cultue: *Trichosporon asahii*) | invasive mechanical ventilation |
| Patient 6 | *Escherichia coli*：urinary infection (urinary culture: *Escherichia coli*) | invasive mechanical ventilation |
| Patient 9 | *Enterococcus faecium:* respiratory infection (respiratory sample culture: *Enterococcus faecium*)  *Candida albicans:* opportunistic infection (respiratory tract, or urinary tract) *HSV-1:* respiratory infection (respiratory mNGS: HSV-1) | *ECMO+* invasive mechanical ventilation |
| Patient 10 | *Candida albicans*: opportunistic infection (respiratory tract, or urinary tract) | *ECMO+* invasive mechanical ventilation |
| Patient 11 | *Candida albicans:* opportunistic infection (respiratory tract, or urinary tract) *Klebsiella pneumoniae:* respiratory infection (respiratory sample culture: *Klebsiella pneumoniae*) | *ECMO+* invasive mechanical ventilation |
| Patient 12 | *HHV-6*: respiratory infection (respiratory mNGS: HHV-6) | invasive mechanical ventilation |
| Patient 22 | *Klebsiella pneumoniae*: respiratory infection (respiratory sample culture: *Klebsiella pneumoniae*) | invasive mechanical ventilation |
| Patient 23 | *Klebsiella pneumoniae*: respiratory infection (respiratory sample culture: *Klebsiella pneumoniae*) | invasive mechanical ventilation |
| Patient 24 | *Escherichia coli*: respiratory infection (respiratory sample culture: *Escherichia coli*) | invasive mechanical ventilation |

***Were all 9 (of 10 non secondary infection patients) patients discharged fromhospital within 60 days, just severe COVID cases? Were all 4 that died, critically ill?***

Thanks for the reviewer’s question. 9 of 10 non secondary infection patients discharged from hospital within 60 days were severe COVID cases. all 4 that died were critically ill patients.

***It would be clearer if the source/site of pathogens detected is included: columns for respiratory, blood and urine***

Thanks for the reviewer’s suggestion. We have listed the table as followed:

|  | **respiratory samples culture** | **respiratory samples mNGS (extra detected)** | **blood samples culture** | **blood samples mNGS (extra detected)** | **Urine samples** |
| --- | --- | --- | --- | --- | --- |
| **Patient 1** | *Acinetobacter baumannii;* |  |  | *HSV-2* |  |
| **Patient 2** | *Enterococcus faecium;Enterococcus faecalis;Aspergillus Fumigatus* | *HSV-1; Staphylococcus spp.* | *Staphylococcus haemolyticus；Candida albicans; Cryptococcus spp.* | *HSV-1* |  |
| **Patient 3** | *Staphylococcus spp.; Enterococcus faecium* | *Klebsiella pneumoniae; Acinetobacter baumannii* |  | *EBV* |  |
| **Patient 4** | *Acinetobacter baumannii;Enterococcus faecium* | *Klebsiella pneumoniae; Stenotrophomonas maltophilia* |  | *Candida albicans* |  |
| **Patient 5** |  | *HSV-1* | *Trichosporon asahii* |  |  |
| **Patient 6** | *Enterococcus faecium ;Klebsiella pneumoniae; Escherichia coli* |  | *Escherichia coli* |  | *Escherichia coli; Candida parapsilosis* |
| **Patient 7** | *Klebsiella pneumoniae; Chryseobacterium spp.;Acinetobacter baumannii* | *HSV-1; Enterococcus faecium* |  |  | *Acinetobacter baumannii* |
| **Patient 8** | *Enterococcus faecium; Penicillium spp.; Acinetobacter baumannii* | *Klebsiella pneumoniae; Mycobacterium intracellulare* |  |  |  |
| **Patient 9** | *Enterococcus faecium; Acinetobacter baumannii* | *HSV-1* | *Enterococcus faecium；Candida albicans* | *HSV-1* |  |
| **Patient 10** | *Enterococcus faecium;Klebsiella pneumoniae; Pseudomonas aeruginosa;Stenotrophomonas maltophilia* | *Mycoplasma hominis* | *Candida albicans* |  | *Candida albicans* |
| **Patient 11** | *Klebsiella pneumoniae; Enterococcus faecium* | *HSV-1* | *Candida albicans;Klebsiella pneumoniae* | | *Candida glabrata* |
| **Patient 12** |  | *HHV-6* | *Staphylococcus haemolyticus；* | *HHV-6* | *Candida albicans; Escherichia coli;  Klebsiella pneumoniae;  Pseudomonas aeruginosa* |
| **Patient 13** | *Aspergillus spp.* |  |  |  | *Escherichia coli* |
| **Patient 14** |  |  |  |  | *Enterococcus faecium* |
| **Patient 15** | *Staphylococcus spp* |  |  |  |  |
| **Patient 17** | *Klebsiella pneumoniae; Pseudomonas aeruginosa;Acinetobacter baumannii* |  |  |  |  |
| **Patient 18** | *Aspergillus spp.* |  |  |  |  |
| **Patient 20** | *Klebsiella pneumoniae* |  |  |  |  |
| **Patient 21** | *Klebsiella pneumoniae* |  |  |  |  |
| **Patient 22** | *Klebsiella pneumoniae* |  | *Klebsiella pneumoniae* |  |  |
| **Patient 23** | *Klebsiella pneumoniae* |  | *Klebsiella pneumoniae* |  |  |
| **Patient 24** | *Acinetobacter baumannii* |  | *Escherichia coli* |  |  |

***Responses to the Reviewer 4:***

***Question 1：The definition of*** ***secondary infection is ambiguous; the authors should clarify the diagnostic criteria of secondary infection for the COVID-19 patients.***
Thanks for the reviewer’s suggestion. We defined the COVID-19 patients who

had clinical symptoms presenting infections or positive radiologic evidence, and a positive laboratory-confirmed etiologic result (culture positive or mNGS positive confirming by RT-PCR) after 48 hours of admission as the one who had secondary infection.

***Question 2. There are too few total cases, it may be the major reason with a high proportion of secondary infection, and It's easy to mislead readers about COVID-19.***

Thank you for your suggestions. We have enrolled 10 more patients into this study and the overall secondary infection rate is 57.89% (22/38).

Co-infections have been described in 14-50% ICU patients [1-4] and bacterial/fungal infection was reported in 8% patients during admission [5].

Yang et al. targeted on 52 critically ill patients with SARS-CoV-2 pneumonia in Wuhan and demonstrated that hospital-acquired infection occurred in seven (13.5%) patients and finally four of them deceased [1]. The pathogens included gram-negative bacteria and fungi [1]. Zhou et al. reported a 50% secondary infection rate in non-survivors (27/54) and ventilator-associated pneumonia occurred in 31% (10/32) patients requiring invasive respiratory support [2].

Compared to previous literatures, we reported a bit higher secondary -infection rate while the number of reported enrolled patients were all 30 to 60. In this study, we sent respiratory, blood and urinary samples timely and performed mNGS for extra detection. Apart from pulmonary infection reported in the two mentioned papers, we analyzed blood-stream infection and urinary infections. We suppose it might lie in **patient characteristics (many of our patients are critically ill or in severe state), repeated culture of multiple samples, and extra detection of viruses and other pathogens through mNGS.**

*[1] X. Yang, Y. Yu, J. Xu, H. Shu, J. Xia, H. Liu, Y. Wu, L. Zhang, Z. Yu, M. Fang, T. Yu, Y. Wang, S. Pan, X. Zou, S. Yuan, Y. Shang, Clinical course and outcomes of critically ill patients with SARS-CoV-2 pneumonia in Wuhan, China: a single-centered, retrospective, observational study, Lancet Respir Med (2020).*

*[2]* *F. Zhou, T. Yu, R. Du, G. Fan, Y. Liu, Z. Liu, J. Xiang, Y. Wang, B. Song, X. Gu, L. Guan, Y. Wei, H. Li, X. Wu, J. Xu, S. Tu, Y. Zhang, H. Chen, B. Cao, Clinical course and risk factors for mortality of adult inpatients with COVID-19 in Wuhan, China: a retrospective cohort study, The Lancet 395(10229) (2020) 1054-1062.*

*[3]* *Arentz M, Yim E, Klaff L, et al. Characteristics and Outcomes of 21 Critically Ill Patients With COVID-19 in Washington State. JAMA 2020;*

*[4] Yang X, Yu Y, Xu J, et al. Clinical course and outcomes of critically ill patients with SARS-CoV-2 pneumonia in Wuhan, China: a single-centered, retrospective, observational study. Lancet Respir. Med. 2020; 8:475–481.*

*[5] Rawson TM, Moore LSP, Zhu N, et al. Bacterial and fungal co-infection in individuals with coronavirus: A rapid review to support COVID-19 antimicrobial prescribing. Clin Infect Dis. 2020 May 2.*

***Question 3. There is too much difference with the recent literature which reported the secondary infection about critical ill COVID-19 patients, and their reported COVID-19 patients seems to be more serious than that in this study, therefore the data in the study is hard to tell the truth of secondary infection.***

Thank you for your suggestions. As mentioned in the question 2, Compared to previous literatures, we reported a bit lower rate of severe and critical patients. It might lie in that cities confront different situations of treating and preventing COVID-19 because of different medical resources, patient numbers and treatment options. In shanghai, we performed careful treatment and timely examinations for each severe and critical ill patients. We sent respiratory, blood and urinary samples timely and performed mNGS for extra detection, thus we believed all the results are convincible to reveal secondary infections of multiple places in these COVID-19 patients.
